# Twenty-Year Public Health Impact of 7- and 13-Valent Pneumococcal Conjugate Vaccines in US Children

**DOI:** 10.3201/eid2706.204238

**Published:** 2021-06

**Authors:** Matt Wasserman, Ruth Chapman, Rotem Lapidot, Kelly Sutton, Desmond Dillon-Murphy, Shreeya Patel, Erica Chilson, Vincenza Snow, Raymond Farkouh, Stephen Pelton

**Affiliations:** Pfizer Inc., New York, New York, USA (M. Wasserman, E. Chilson, V. Snow, R. Farkouh);; Evidera Market Access Ltd, London, UK (R. Chapman, K. Sutton, D. Dillon-Murphy, S. Patel);; Boston University School of Medicine, Boston, Massachusetts, USA (R. Lapidot, S. Pelton)

**Keywords:** children, incidence, invasive pneumococcal disease, IPD, otitis media, pneumococcal disease, pneumococcal vaccination, PCV7, PCV13, pneumococcal pneumonia, literature review, United States, vaccination, vaccines, vaccine-preventable diseases, bacteria, bacterial infections, PCVs, pneumococcal conjugate vaccines, *Streptococcus pneumoniae*

## Abstract

Pneumococcal conjugate vaccines (PCVs) have been used in the United States since 2000. To assess the cumulative 20-year effect of PCVs on invasive pneumococcal disease (IPD) incidence among children <5 years of age, we analyzed Active Bacterial Core Surveillance data, conducted a literature review, and modeled expected and observed disease. We found that PCVs have averted >282,000 cases of IPD, including ≈16,000 meningitis, ≈172,000 bacteremia, and ≈55,000 bacteremic pneumonia cases. In addition, vaccination has prevented 97 million healthcare visits for otitis media, 438,914–706,345 hospitalizations for pneumonia, and 2,780 total deaths. IPD cases declined 91%, from 15,707 in 1997 to 1,382 in 2019. Average annual visits for otitis media declined 41%, from 78 visits/100 children before PCV introduction to 46 visits/100 children after PCV13 introduction. Annual pneumonia hospitalizations declined 66%–79%, from 110,000–175,000 in 1997 to 37,000 in 2019. These findings confirm the substantial benefits of PCVs for preventing IPD in children.

Before 2000, children <2 years of age had the highest incidence of invasive pneumococcal diseases (IPDs) such as bacteremia, meningitis, or other infection of a normally sterile site (*1*). Researchers estimated that, in the United States, annual IPD incidence was 165 cases/100,000 children <12 months of age and 203 cases/100,000 children 12–23 months of age (*1*). Until the United States began a universal 7-valent pneumococcal conjugate vaccine (PCV7) immunization program for children in 2000, *Streptococcus pneumoniae* was the leading cause of bacterial meningitis (*1*). *S. pneumoniae *was also the most common bacterial cause of community-acquired pneumonia and otitis media (OM) in young children. Furthermore, in the 1990s, concerns emerged regarding the growing number of pneumococcal isolates with reduced susceptibility to first- and second-line antimicrobial drugs (*1*).

PCV7 was the first pneumococcal conjugate vaccine (PCV) approved for use in children <2 years of age in the United States. Pneumococcal polysaccharide vaccines, which preceded PCVs, are not immunogenic in children <2 years of age (*1*,*2*). PCV7 overcame the challenge of poor immunogenicity among infants and young children through conjugation technology; it was introduced into the US infant immunization schedule in 2000, providing direct protection against several serotypes of invasive and noninvasive pneumococcal disease (*3*,*4*). PCV7 protects against the *S. pneumoniae* serotypes responsible for >80% of IPD cases among children in North America (i.e., serotypes 4, 6B, 9V, 14, 18C, 19F, and 23F) (*1*,*4*). In 2010, PCV13, a vaccine providing protection against 6 additional serotypes (i.e., serotypes 1, 3, 5, 6A, 19A, and 7F), was approved in the United States, partially because of increasing incidence of serotypes not covered by PCV7 (*1*,*4*).

Clinical trial data suggested that PCV7 would be effective against IPD, OM, and according to a post-hoc analysis, pneumonia (*5*). The efficacy of PCV7 (and later PCV13) against all forms of pneumococcal disease was greater than expected, partly because of indirect protection gained through herd immunity (*6*–*8*). The United States was the first country to introduce a PCV program for infants and, during the transition to PCV13, recommended the largest catch-up program for children <5 years of age who had been vaccinated with PCV7 (*9*). After an initially slow uptake limited by constrained supply, the United States has achieved consistently high (>80%) 3-dose coverage since 2005 (*10*). It is one of a few countries continuing to use the licensed 4-dose schedule (*10*). A 2020 review demonstrated that PCVs were the only vaccines approved by the US Food and Drug Administration that had no postmarketing safety-related label modifications (*11*).

We quantified the decrease of IPD incidence associated with 20 years of PCV use in the United States. First, we conducted a literature review to inform a decision analytic model. The model estimated the 20-year cumulative effects associated with the PCV program on cases of IPD, OM, and hospitalizations for pneumonia among children <5 years of age in the United States.

## Methods

The US Centers for Disease Control and Prevention (CDC) began the Active Bacterial Core Surveillance (ABCs) program to monitor invasive *S. pneumoniae* infections in 1997 (*12*). Although this resource provides invaluable data for assessing IPD, it does not include data on noninvasive syndromes. We conducted a literature review to identify and synthesize published data on all pneumococcal diseases during the past 20 years. We used data from these publications to model the effects of PCVs on childhood pneumococcal disease (*13*).

### Literature Review

To estimate the amount of pneumococcal disease averted in the United States, we conducted a systematic literature review in accordance with the Preferred Reporting Items for Systematic Reviews and Meta-Analyses guidelines (*14*). After defining the research questions, data sources, search strategies, and selection criteria (Appendix Tables 1–5), we conducted electronic searches of the PubMed and Embase (https://www.embase.com) databases and manual searches of the gray literature, CDC website (https://www.cdc.gov), and reference lists of 7 published literature reviews (*15*–*21*) (Appendix Figure 1). This study describes only references used for input data or to validate our findings.

### Calculations and Outputs

We developed a model using Excel (Microsoft, https://www.microsoft.com) to calculate the national numbers of cases, healthcare visits, hospitalizations, and deaths caused by pneumococcal infection among children <5 years of age during the 20 years after PCV introduction. We used published incidences of each syndrome (i.e., meningitis, bacteremia, bacteremic pneumonia/empyema, sepsis, and other) and relevant population data to calculate the number of cases averted by vaccination. We conducted these calculations for the pre-PCV (i.e., 1997–1999), PCV7 (i.e., 2000–2009), and PCV13 (i.e., 2010–2019) eras (Appendix Figure 2). Although we attributed decreasing illness and deaths to the direct effects of PCVs, policy changes or other interventions also might have contributed to the reduction of disease.

**Figure 1:**
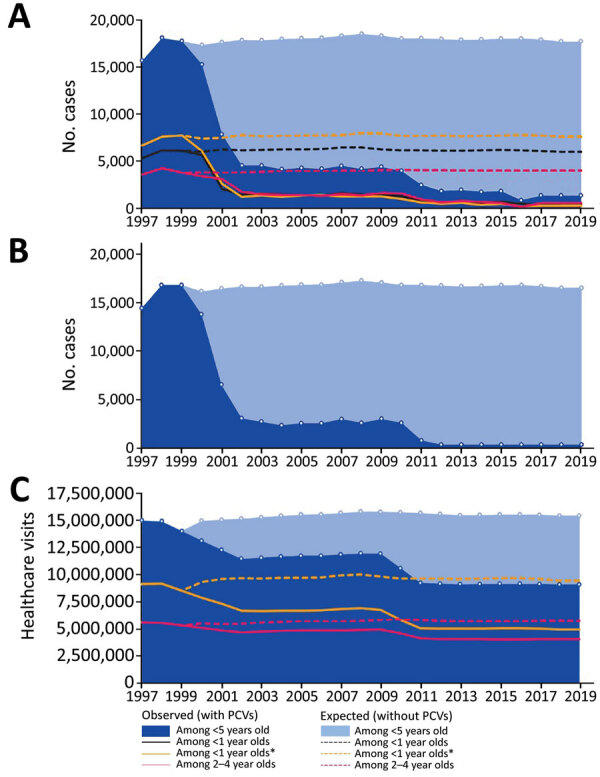
Effects of PCVs on invasive pneumococcal disease (IPD) and otitis media among children <5 years of age, United States, 1997–2019 (*8*,*12*). A) Cases of IPD. B) Cases of IPD caused by 13-valent PCV serotypes. C) Healthcare visits for otitis media. The United States approved 7-valent PCV in 2000 and 13-valent PCV in 2010. Asterisk (*) indicates that for data on healthcare visits for otitis media, age range is 0–2 years. PCV, pneumococcal conjugate vaccine.

**Figure 2:**
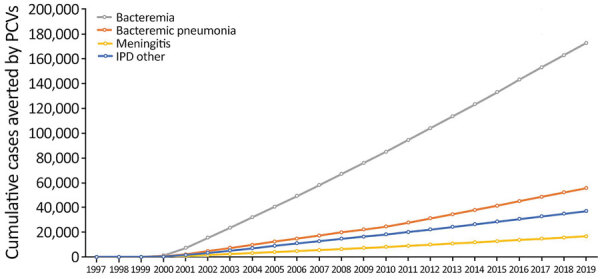
Effects of PCVs on different syndromes of IPD in children <5 years of age, United States, 1997–2019. The United States approved 7-valent PCV in 2000 and 13-valent PCV in 2010. IPD, invasive pneumococcal disease; PCV, pneumococcal conjugate vaccine.

We calculated average incidences for each of the 3 described time periods. Because variance measures were unavailable, we performed all calculations as point estimates. We assumed that without PCVs, disease incidence would have remained constant. We calculated the estimated effect of PCV7 by comparing the difference in reported incidence between the pre-PCV and PCV7 eras; likewise, we considered the effect of PCV13 to be the difference in incidence between the pre-PCV and PCV13 eras. We estimated the incremental effect of including the additional serotypes in PCV13 by comparing incidence between the PCV7 and PCV13 eras. Because factors such as program rollout and uptake delayed the achievement of population-level equilibrium, we excluded the transition years 2000–2001 from the calculation of the effect of PCV7. Similarly, we excluded 2010 from the calculation of the effect of PCV13 (Tables 1, 2; Figures 1, 2). However, we included these years in the analysis of the 20-year aggregate effect of PCV use.

**Table 1:** Invasive pneumococcal disease cases and deaths averted by pneumococcal conjugate vaccines, United States, 1997–2019*

Year	No. observed, by age, y					No. expected, by age, y					No. averted	
	<1	1	2–4	Overall		<1	1	2–4	Overall		Annual	Cumulative
No. cases												
1997	5,360	6,712	3,635	15,707		5,360	6,712	3,635	15,707		NA	NA
1998	6,220	7,630	4,286	18,136		6,220	7,630	4,286	18,136		NA	NA
1999	6,176	7,772	3,837	17,785		6,176	7,772	3,837	17,785		NA	NA
2000	5,699	6,139	3,469	15,306		6,053	7,428	3,883	17,364		2,057	2,057
2001	2,099	2,645	3,143	7,887		6,299	7,539	3,852	17,689		9,802	11,860
2002	1,521	1,261	1,813	4,596		6,202	7,830	3,866	17,899		13,304	25,164
2003	1,642	1,405	1,530	4,577		6,241	7,696	3,936	17,874		13,296	38,460
2004	1,485	1,253	1,454	4,192		6,301	7,730	3,983	18,013		13,821	52,281
2005	1,450	1,419	1,467	4,336		6,286	7,796	4,019	18,101		13,765	66,046
2006	1,366	1,458	1,395	4,219		6,344	7,767	4,019	18,130		13,911	79,956
2007	1,680	1,296	1,560	4,537		6,511	7,826	4,036	18,372		13,835	93,792
2008	1,517	1,284	1,420	4,221		6,487	8,018	4,057	18,562		14,341	108,133
2009	1,485	1,309	1,654	4,449		6,284	7,975	4,099	18,358		13,910	122,043
2010	1,352	1,051	1,611	4,014		6,204	7,726	4,144	18,074		14,060	136,103
2011	832	670	1,012	2,515		6,221	7,755	4,109	18,085		15,571	151,674
2012	616	541	712	1,870		6,163	7,777	4,068	18,009		16,139	167,813
2013	578	595	814	1,988		6,171	7,709	4,036	17,916		15,928	183,741
2014	629	407	754	1,790		6,208	7,721	4,033	17,963		16,173	199,914
2015	733	513	610	1,856		6,253	7,769	4,031	18,053		16,198	216,112
2016	526	212	167	906		6,208	7,828	4,032	18,068		17,163	233,275
2017	452	314	625	1,391		6,112	7,770	4,052	17,934		16,544	249,818
2018	446	309	627	1,382		6,040	7,651	4,061	17,752		16,370	266,188
2019	446	309	627	1,382		6,040	7,651	4,061	17,752		16,370	282,558
No. deaths												
1997	151	34	17	202		151	34	17	202		NA	NA
1998	78	47	NA	126		78	47	NA	126		NA	NA
1999	29	45	60	134		29	45	60	134		NA	NA
2000	130	113	57	301		138	137	64	340		39	39
2001	22	46	57	125		103	66	36	204		79	118
2002	31	NA	32	62		101	68	36	205		143	261
2003	42	21	21	85		102	67	37	206		121	382
2004	39	48	20	107		103	67	37	207		101	483
2005	38	9	38	85		103	68	37	208		123	605
2006	29	9	47	85		104	68	37	209		124	729
2007	37	9	9	56		106	68	37	212		156	885
2008	37	NA	18	55		106	70	38	214		158	1,043
2009	36	9	9	54		103	70	38	210		156	1,199
2010	10	9	9	28		101	67	38	207		179	1,378
2011	38	19	28	85		102	68	38	207		123	1,501
2012	9	9	NA	19		101	68	38	206		187	1,688
2013	9	9	38	57		101	67	37	205		149	1,837
2014	19	NA	9	28		101	67	37	206		178	2,014
2015	10	9	19	38		102	68	37	207		170	2,184
2016	18	4	12	35		101	68	37	207		172	2,356
2017	28	19	38	85		100	68	38	205		120	2,476
2018	16	7	28	51		99	67	38	203		152	2,628
2019	16	7	28	51		99	67	38	203		152	2,780

*Values are rounded to the nearest whole numbers. NA, not applicable.

**Table 2:** Cases of invasive pneumococcal disease averted by PCV13, United States, 1997–2019*

Year	No. observed				No. expected				Cumulative cases averted		Difference in non-PCV13 serotype cases†
	All	PCV13 serotypes	Non-PCV13 serotypes		All	PCV13 serotypes	Non-PCV13 serotypes		All	PCV13 serotypes	
1997	15,543	14,439	1,104		15,543	14,439	1,104		NA	NA	NA
1998	18,136	16,848	1,288		18,136	16,848	1,288		NA	NA	NA
1999	17,785	16,839	945		17,785	16,839	945		NA	NA	NA
2000	15,306	13,808	1,498		17,364	16,178	1,186		2,057	2,369	−312
2001	7,887	6,561	1,325		17,689	16,481	1,208		11,860	12,289	−429
2002	4,596	3,109	1,487		17,899	16,677	1,223		25,164	25,857	−693
2003	4,577	2,743	1,834		17,874	16,653	1,221		38,460	39,767	−1,307
2004	4,192	2,374	1,818		18,013	16,783	1,230		52,281	54,175	−1,894
2005	4,336	2,589	1,747		18,101	16,864	1,236		66,046	68,450	−2,405
2006	4,219	2,592	1,627		18,130	16,891	1,238		79,956	82,750	−2,793
2007	4,537	3,019	1,518		18,372	17,118	1,255		93,792	96,848	−3,057
2008	4,221	2,635	1,585		18,562	17,294	1,268		108,133	111,507	−3,374
2009	4,449	3,037	1,412		18,358	17,104	1,254		122,043	125,575	−3,532
2010	4,014	2,626	1,388		18,074	16,839	1,235		136,103	139,788	−3,685
2011	2,515	805	1,710		18,085	16,850	1,235		151,674	155,833	−4,160
2012	1,870	400	1,470		18,009	16,779	1,230		167,813	172,213	−4,400
2013	1,988	397	1,591		17,916	16,692	1,224		183,741	188,508	−4,766
2014	1,790	397	1,392		17,963	16,736	1,227		199,914	204,846	−4,932
2015	1,856	398	1,457		18,053	16,820	1,233		216,112	221,268	−5,156
2016	906	398	507		18,068	16,834	1,234		233,275	237,703	−4,429
2017	1,391	398	993		17,934	16,709	1,225		249,818	254,015	−4,197
2018	1,382	396	986		17,752	16,539	1,213		266,188	270,158	−3,970
2019	1,382	396	986		17,752	16,539	1,213		282,558	286,302	−3,744

*Values are rounded to the nearest whole numbers. NA, not applicable; PCV13, 13-valent pneumococcal conjugate vaccine.  †Negative values indicate greater number observed than would be expected.

We calculated the number of expected IPD cases without PCV7 as the average incidence during 1997–1999 × population size in each year. We calculated the expected IPD cases if PCV7 vaccination had continued but PCV13 had not been introduced as the average incidence during 2002–2009 × population size in each year. We stratified each calculation by age.

In addition, we calculated total IPD cases averted by PCVs as the difference between the cases expected without vaccination and the cases observed during 2002–2019 (Table 1; Appendix Figure 2). We also calculated the incremental effect of PCV13 versus PCV7 as the difference between cases expected if PCV7 use had continued after 2010 and cases observed during 2011–2019.

To calculate the number of expected IPD deaths without PCVs, we multiplied the observed case-fatality ratio from 1997–2000 (cumulative deaths divided by the cumulative cases in that period) by the expected number of cases from 2000–2019 (*12*). We considered deaths averted by PCVs to be the difference between expected deaths if PCVs had never been introduced and the observed deaths in this period. 

Case numbers and incidences were not available for OM and noninvasive pneumonia because they are nonnotifiable diseases. As a result, we calculated the expected ambulatory healthcare visit rates for OM and hospitalization rates for pneumonia without PCVs using the same method as for IPD cases averted.

### Model Inputs

We conducted our calculations using population data from the US Census Bureau (*22*) (Appendix Table 6). We considered data on total IPD incidence, distribution of vaccine serotypes, syndrome distribution, healthcare visits for OM, and pneumonia incidence.

We obtained national estimates for IPD cases, rates, and syndromes among children <1, 1–<2, and 2–4 years of age from ABCs reports (*12*) (Appendix Table 7). Because data for 2018 and 2019 were not available, we assumed these years to have the same rates as 2017. We used these data to calculate the average incidence for each of the 2 pre-PCV13 eras (Appendix Table 8).

We also obtained national estimates for overall annual incidence of IPD caused by PCV13 serotypes among children <5 years of age during 1998–2016 (*12*) (Appendix Table 9). We assumed rates during 2017–2019 to be the same as 2016; we weighted these rates by population distribution during those years. We calculated pre-PCV era distribution of PCV13 and non-PCV13 serotypes as the average of 1997–1999 distributions (Appendix Table 10). To ensure serotype incidences were consistent with the observed trends of all IPDs, we imputed PCV13 serotype incidence in 1997 using weighted proportions (i.e., by population size in each age group) and percent change (Appendix Table 9) during 1997–1998. We calculated expected cases caused by PCV13 and non-PCV13 serotypes by multiplying the average pre-PCV era serotype distributions to the total expected number of annual IPD cases. Although the measurement of all averted cases of IPD includes the effects of vaccination and serotype replacement, the measurement of cases averted by PCV13 indicates only the reduction in vaccine type IPDs.

We obtained the proportions of meningitis, bacteremia, bacteremic pneumonia/empyema, sepsis, and other infections among children <2 and 2–4 years of age with IPD from additional unpublished data provided by CDC (*12*; R. Gierke, CDC, pers. comm., 2017 Nov 7) (Appendix Table 11). We assumed the distributions in 1997–1999 to be the same as 2000.

We used the mean rate of ambulatory care visits for OM in children <2, 2–<5, and <5 years of age overall provided by Zhou et al. (*23*; Appendix Table 12, Figure 3). Zhou et al. (*23*) described PCV eras using similar definitions: the pre-PCV period during 1997–1998, the PCV7 period during 2002–2009, and the PCV13 period during 2011–2013 (Appendix Table 13). We assumed the rates in 2014–2019 to be the same as 2013 (Appendix Table 12).

The data sources used various classifications and definitions of pneumonia. The types of data reported also varied widely, including measurements such as ambulatory visits, hospitalizations, index cases in inpatients, and estimates of cases of community-acquired pneumonia. No single data source covered the combined PCV7 and PCV13 periods, nor estimated the incidence of only noninvasive pneumococcal pneumonia. Because hospitalization data represent more severe cases with the largest use of healthcare costs and resources and because no consistent data for ambulatory/outpatient visits for pneumonia during the entire study period were available, we considered only hospitalized cases of pneumonia in this analysis. We used data on hospitalization for pneumonia from multiple sources. We obtained data for the pre-PCV relative to PCV7 eras from Simonsen et al. (*24*), Foote et al. (*25*), and Grijalva et al. (*26*), and for the PCV7 relative to PCV13 period from a 2005–2014 study (*27*) and Tong et al. (*28*). In addition, we used estimates of the difference in hospitalization incidences during the PCV7 period from Grijalva et al. (*23*). We estimated the total number of hospitalizations averted by PCVs using the expected hospitalization data from all sources for 1997–2019 (Appendix Table 14).

### Validation

During the literature review, we identified appropriate references against which to validate the consistency of our findings. We did not identify other sources of national multistate data for IPD comparable to the ABCs dataset. Black et al. (*30*) reported Kaiser Permanente data from northern California about the effect of PCV7 on disease epidemiology in children and adults, whereas Yildirim et al. (*31*) reported serotype-specific invasive capacity among children in Massachusetts after PCV introduction (Appendix Figure 4).

We used a large national claims database (*32*), a commercial claims and encounters database (*33*), and Ray et al. (*34*) to validate our OM estimates. We wanted to validate our pneumonia estimates with respect to different types of cases and definitions used by various data sources; however, because of constraints on data availability, we limited our validation to hospitalized cases of all-cause pneumonia. Because of the variation in reporting of pneumonia data, we did not identify any alternate sources for an appropriate validation of our analysis.

## Results

### IPD

Among children <5 years of age, the annual number of IPD cases decreased from ≈16,000−18,000 during 1997−1999 to 1,382 in 2019 (Table 1; Figure 1, panel A). We estimated that PCVs averted a cumulative 282,558 cases of IPD during this timeframe. Of those averted cases, we estimated that 146,455 were prevented by PCV13 during 2010–2019. Among children <5 years of age, annual deaths caused by IPD decreased from 126−202 during 1997−1999 (Table 1) to 85 in 2019. We estimated that PCVs prevented a total of 2,780 deaths during this timeframe, including 1,402 deaths prevented by PCV13 during 2010–2019.

The overall IPD incidences in the input ABCs data were generally higher than in the sources used for validation. However, the ABCs and the Kaiser Permanente data (*30*) reflected similar overall trends for the pre-PCV and PCV7 eras; the ABCs and data from Yildrim et al. (*31*) reflected similar overall trends for the PCV7 and PCV13 eras. The differences were probably caused by variations in reporting and patient groups between data sources; the ABCs data are more nationally representative and therefore more generalizable than the population described by Black et al. (*30*).

We observed a decrease in IPD-related deaths after the introduction of PCV7; however, we could not identify whether this trend existed during the pre-PCV period because of limited data (Table 1). Pulido et al. (*35*) described a declining IPD mortality rate during 1990–2005, supporting the ABCs data and indicating that deaths were already decreasing before the introduction of PCVs. Reductions in smoking rates and implementation of laws regarding smoking in public places, shifts from inpatient to outpatient care settings, improved treatments, and varying case definitions might have also contributed to the declining trend.

We estimated that during 2000–2019, PCVs prevented 172,778 cases of bacteremia; 55,532 cases of bacteremic pneumonia and empyema; 16,660 cases of meningitis; and 37,017 cases of other forms of IPD (Figure 2). IPD cases caused by PCV13 serotypes decreased from 14,439 cases in 1997 to 396 in 2019 (Table 2; Figure 1, panel B). During 2000–2019, PCVs are estimated to have averted 286,302 IPD cases caused by vaccine serotypes. During this period, IPD cases caused by non-PCV13 serotypes increased slightly, consistent with modest serotype replacement.

### OM Healthcare Visits

The average rate of OM visits among children <5 years of age declined from 78/100 to 46/100 children per year from the pre-PCV (1997–1998) to the PCV13 era (2011–2013). In other words, these visits declined by 39%, from 15,000,483 in 1997 to 9,112,727 in 2019 (Figure 1, panel C). We estimated that PCVs averted a cumulative 97,326,688 OM-related healthcare visits (Table 3).

**Table 3:** Estimated average incidence of otitis media cases and visits averted by PCVs, United States, 1997–2019*

Measure	1997–1999	2000–2009	2010–2019	Cumulative
Average incidence of visits per 100 children	78	59	46	NA
Expected visits†	NA	154,269,900	155,511,917	309,781,817
Estimated visits	NA	119,429,938	93,025,190	212,455,128
Visits averted‡	NA	34,839,962	62,486,726	97,326,688

*Values are rounded to the nearest whole numbers. PCV7 was approved for use in the United States in 2000; PCV13 was approved for use in the United States in 2010. NA, not applicable; PCV, pneumococcal conjugate vaccine; PCV7, 7-valent PCV; PCV13, 13-valent PCV. †Visits expected if PCVs had not been introduced. ‡Calculated by subtracting estimated visits from expected visits.

The overall visit numbers in the input data (*23*) were generally lower than in the sources used for validation (*32*–*34*), but the overall trends for the pre-PCV and PCV7 eras were comparable. The differences might have been caused by varying database populations, because Zhou et al. (*23*) used national data whereas Marom et al. (*32*) and Tong et al. (*33*) mainly considered privately insured patients who might have been more likely to seek care. Therefore, the estimates from Zhou et al. (*23*) are probably more representative on a national level.

### Pneumonia Hospitalizations

Annual pneumonia hospitalizations in children <5 years of age declined from 113,116–175,420 in 1997 to 37,882 in 2019. We estimated that PCVs averted a cumulative 438,914–706,345 pneumonia hospitalizations, including 216,303 cases caused by PCV13 serotypes, during the 20 years after PCV introduction in the United States (Table 4).

**Table 4:** Estimated total hospitalized cases of pneumonia averted by PCVs, United States, 1997–2019*

Time period	Estimated hospitalizations					Total hospitalizations averted	
	Observed, with vaccination			Expected without vaccination			
	Minimum	Maximum		Minimum	Maximum	Minimum	Maximum
Pre-PCV era: 1997–1999	NA	NA		339,474	525,675	NA	NA
PCV7 era: 2000–2009†	959,543	1,336,673		597,479	1,822,591	222,611	490,043‡
PCV13 era: 2010–2019§	382,182			598,484		216,303	

*PCV7 was approved for use in the United States in 2000; PCV13 was approved for use in the United States in 2010. NA, not applicable; PCV, pneumococcal conjugate vaccine; PCV7, 7-valent PCV; PCV13, 13-valent PCV. †Averted hospitalizations during PCV7 era are shown as the difference within the same study with minimum based on Simonson et. al. (*24*) and maximum based on Grijalva et al (*29*) (Appendix Table 14). ‡Grijalva et al (*29*) reported a change in hospitalization rate; as a result, no observed and expected values were generated. §Values based on calculations using a single data point.

## Discussion

Vaccines, especially PCVs, are lifesaving and cost-effective public health interventions. At the time PCV7 was introduced in the United States, pneumococcal disease caused high rates of death and disease among infants. Despite conservative findings from the clinical trials (*8*), public health officials and healthcare professionals were optimistic about the potential of PCVs to prevent pneumococcal disease. We conducted a literature review and modeling analysis to quantify the effects of PCVs on pneumococcal disease incidence among children <5 years of age, a population at higher risk for IPD and therefore the focus of IPD prevention efforts (*36*). Our analysis demonstrated that PCVs have averted >282,000 cases of IPD and 2,780 associated deaths, with reductions across various IPD syndromes. Since their introduction in 2000, PCVs have averted >430,000 pneumonia hospitalizations and >97,000,000 OM-related healthcare visits. 

We could not find data on all OM cases; our analysis instead measured ambulatory care visits using input data from Zhou et al. (*23*). However, because not all children with OM receive treatment through ambulatory care visits, our findings probably underestimate the true effects of PCVs on OM incidence. The annual number of OM visits declined from 78 visits/100 to 46 visits/100 children from the pre-PCV era (1997–1999) to PCV13 era (2010–2019); this 41% decline exceeds the original predictions based on early clinical trial data (*8*). Vaccination is probably the main direct contributor to this reduction; however, vaccination also might have had indirect effects such as changes in the disease definition or clinical coding of OM, as well as changes in prescribing patterns of antimicrobial drugs, which might affect healthcare use. Although not all cases of OM prompt healthcare visits, even mild illnesses might require family members to take time from work to care for their children, further reducing productivity and quality of life in ways not reflected by this metric (*37*).

Because we could not find data on noninvasive pneumonia, we combined multiple data sources to compare pneumonia hospitalizations during the study period. These data are probably underestimates of the true effect of PCVs because most pneumonia cases among children <5 years of age do not require hospitalization.

The overall findings are impressive but nevertheless conservative. We did not consider the direct benefits of reduced sequelae among children >5 years of age nor adults; we also did not consider indirect benefits such as herd immunity, reduced use of antimicrobial drugs and other healthcare resources, increased educational attainment, or improved parental productivity. In addition, we did not analyze data on common but less resource-intensive manifestations of *S. pneumoniae* such as conjunctivitis (*38*,*39*). Finally, this analysis does not reflect PCVs’ effects on antimicrobial resistance (*17*), although preventing infection through vaccination reduces the need for antimicrobial treatment (*40*).

In agreement with other studies (*40*,*41*), we found that IPD cases caused by PCV13 serotypes declined while the number of cases caused by non-PCV13 serotypes slightly increased, reflecting modest serotype replacement. PCVs have been used in the United States longer than in any other country. Because of the large quantity of available data, we conducted a literature review to independently identify appropriate references for model inputs and validation. However, although the available data were extensive, it was not comprehensive. As a result, this research was limited by the lack of a single data source for pneumonia and OM incidences during the entire study period, prompting us to impute values for years when no data were available. In addition, because IPD is a notifiable disease but OM and pneumonia are not, we can only estimate PCVs’ true effects on OM and pneumonia incidence using alternative metrics such as ambulatory care visits and hospitalizations. Furthermore, healthcare providers do not usually distinguish the causative bacteria of pneumonia and OM cases, which poses difficulties in analyzing serotype distributions. Finally, we could not find alternative national-level IPD data for the validation analysis, prompting us to compare our results with trends from smaller regions.

 CDC and the Advisory Committee on Immunization Practices have recommended the use of PCVs in a national infant immunization program since 2000 (*1*). Our model used available data to quantify the effects of PCV7 and PCV13 on pneumococcal disease burden among children in the United States. Our results demonstrate the effectiveness of PCVs in preventing illness and death among children <5 years of age.

Appendix Further information on effects of 7- and 13-valent pneumococcal conjugate vaccines on disease in children, United States, 1997–2019.
