# Supplementary material for: Twenty-Year Public Health Impact of 7- and 13-Valent Pneumococcal Conjugate Vaccines in US Children
Source: Emerg Infect Dis. 2021 Jun;27(6):1627–36. doi: 10.3201/eid2706.204238 (PMC8153862; doi:10.3201/eid2706.204238)
Supplement: Appendix — Further information on effects of 7- and 13-valent pneumococcal conjugate vaccines on disease in children, United States, 1997–2019. [file 20-4238-Techapp-s1.pdf]

Article DOI: https://doi.org/10.3201/eid2706.204238

Twenty-Year Public Health Impact of 7- and 13-Valent Pneumococcal Conjugate Vaccines in US Children

Appendix

Literature Review

Our searches were limited to English language articles involving human participants published during 1999–2019. After identifying duplicate results, we screened abstracts against the prespecified patient, intervention, comparison, outcome, and study-based eligibility criteria (Appendix Table 4). We obtained full-text references for the eligible articles for independent screening, yielding 167 articles: 96 full-text and 71 gray literature publications (Appendix Figure 1). We reviewed these articles against additional inclusion limits (Appendix Table 5); these limits considered US Food and Drug Administration–approved label indications for PCVs but included all outcome measures describing differences in clinical incidence since 2000, when PCV7 was introduced in the United States. Data from the final 125 sources were extracted into a bespoke extraction template by a single investigator. All data were validated by a 2nd investigator, with any discrepancies resolved by a 3rd, senior investigator.

Appendix Table 1. Data sources and search limits in study on pneumococcal conjugate vaccines

Domain	Scope
Data sources	
Electronic databases	Embase
	Medline (including in-process articles)
Gray literature*	International Symposium on Penumoccoci & Penumoccocal Diseases 2018
	IDWeek 2017 and 2018
	European Society for Paediatric Infectious Diseases 2018 and 2019
	US Centers for Disease Control and Prevention†
Search criteria	
Publication date	1999–2019
Geographic region	United States
Language	English

*Manual search of abstracts from conference websites (https://isppd2018.kenes.com/2018/Pages/default.html, https://idweek.org/events/idweek2017, https://idweek.org/events/idweek2018, https://espidmeeting.org/past-espid-abstracts)
†(*12*).

Appendix Table 2. Medline search algorithm in study on pneumococcal conjugate vaccines

Search	Terms	Results*
1	“Pneumococcal Vaccines” [mesh] OR “Heptavalent Pneumococcal Conjugate Vaccine” [mesh] OR “13-valent pneumococcal vaccine”[tiab] OR “prevenar13”[tiab] OR “PCV13 vaccine”[tiab] OR “PCV-13 vaccine”[tiab] OR “prevenar 13”[tiab] OR “prevnar 13”[tiab] OR “moniarix”[tiab] OR “synflorix”[tiab] OR “PHiD-CV vaccine”[tiab] OR “23-valent vaccine”[tiab] OR “23-valent pneumococcal capsular polysaccharide vaccine”[tiab] OR “Pneumovax 23”[tiab] OR “PPSV23”[tiab] OR “Pneumo-23”[tiab] OR “10-valent pneumococcal conjugate vaccine”[tiab] OR “ten-valent PCV”[tiab] OR “PCV10”[tiab] OR “streptopur”[tiab]	7,310
2	“burden”[tiab] OR “incidence”[tiab] OR “prevalence”[tiab] OR “impact”[tiab] OR “effectiveness”[tiab] OR “mortality”[tiab]	2,927,356
3	“otitis media”[tiab] OR “pneumonia”[tiab] OR “pneumococcal disease”[tiab] OR “invasive pneumococcal disease”[tiab] OR “meningitis”[tiab]	179,808
4	Results of search no. 2 OR 3	3,059,509
5	“united states” [tiab] or “north america”[tiab]	262,751
6	“clinical trial”[pt] OR “controlled clinical trial”[pt] OR “model”[tiab] “practice guideline”[pt] OR “controlled trial”[tiab]	117,526
7	Results of search no. 1 AND 4 AND 5 NOT 6	290
8	Results of search no. 7 + filters: publication date after 1999 Jan 1; English language	266

*As of 2019 May 21.

Appendix Table 3. Embase search algorithm in study on pneumococcal conjugate vaccines

Search	Terms	Results*
1	('pneumococcus vaccine'/exp OR 'pneumococcus vaccine' OR 'heptadecavalent pneumococcal vaccine'/exp OR 'heptadecavalent pneumococcal vaccine' OR 'heptadecavalent pneumococcus vaccine'/exp OR 'heptadecavalent pneumococcus vaccine' OR 'heptavalent pneumococcal conjugate vaccine'/exp OR 'heptavalent pneumococcal conjugate vaccine' OR 'moniarix'/exp OR 'moniarix' OR 'pcv 13'/exp OR 'pcv 13' OR 'pcv13'/exp OR 'pcv13' OR 'phid cv.'/exp OR 'phid cv.' OR 'phid cv. vaccine'/exp OR 'phid cv. vaccine' OR 'pneu immune'/exp OR 'pneu immune' OR 'pneumo 23'/exp OR 'pneumo 23' OR 'pneumo 23 imovax'/exp OR 'pneumo 23 imovax' OR 'pneumococcal 10-valent conjugate vaccine'/exp OR 'pneumococcal 10-valent conjugate vaccine' OR 'pneumococcal 13-valent conjugate vaccine'/exp OR 'pneumococcal 13-valent conjugate vaccine' OR 'pneumococcal 14-valent conjugate vaccine'/exp OR 'pneumococcal 14-valent conjugate vaccine' OR 'pneumococcal 23-valent conjugate vaccine'/exp OR 'pneumococcal 23-valent conjugate vaccine' OR 'pneumococcal 7-valent conjugate vaccine'/exp OR 'pneumococcal 7-valent conjugate vaccine' OR 'pneumococcal conjugate vaccine'/exp OR 'pneumococcal conjugate vaccine' OR 'pneumococcal conjugate vaccine (nos)'/exp OR 'pneumococcal conjugate vaccine (nos)' OR 'pneumococcal polysaccharide conjugate vaccine'/exp OR 'pneumococcal polysaccharide conjugate vaccine' OR 'pneumococcal polysaccharide conjugate vaccine (13 valent, adsorbed)'/exp OR 'pneumococcal polysaccharide conjugate vaccine (13 valent, adsorbed)' OR 'pneumococcal polysaccharide conjugate vaccine (adsorbed)'/exp OR 'pneumococcal polysaccharide conjugate vaccine (adsorbed)' OR 'pneumococcal saccharide conjugated vaccine, adsorbed'/exp OR 'pneumococcal saccharide conjugated vaccine, adsorbed' OR 'pneumococcal vaccine'/exp OR 'pneumococcal vaccine' OR 'pneumococcal vaccines'/exp OR 'pneumococcal vaccines' OR 'pneumococcus polysaccharide vaccine'/exp OR 'pneumococcus polysaccharide vaccine' OR 'pneumopur'/exp OR 'pneumopur' OR 'pneumovax'/exp OR 'pneumovax' OR 'pneumovax 23'/exp OR 'pneumovax 23' OR 'pneumovax ii'/exp OR 'pneumovax ii' OR 'pnu immune'/exp OR 'pnu immune' OR 'pnu imune'/exp OR 'pnu imune' OR 'pnu imune 23'/exp OR 'pnu imune 23' OR 'pnu-imune 23'/exp OR 'pnu-imune 23' OR 'prevenar'/exp OR 'prevenar' OR 'prevenar 13'/exp OR 'prevenar 13' OR 'prevenar13'/exp OR 'prevenar13' OR 'prevnar'/exp OR 'prevnar' OR 'prevnar 13'/exp OR 'prevnar 13' OR 'streptococcus pneumoniae vaccine'/exp OR 'streptococcus pneumoniae vaccine' OR 'streptopur'/exp OR 'streptopur' OR 'streptorix'/exp OR 'streptorix' OR 'synflorix'/exp OR 'synflorix')	19,499
2	'burden':ab,ti OR ‘impact’:ab,ti OR ‘incidence’:ab,ti OR ‘prevalence’:ab,ti OR ‘effectiveness’:ab,ti OR ‘mortality’:ab,ti	4,078,907
3	'otitis media':ab,ti OR 'pneumonia':ab,ti OR ‘pneumococcal disease’:ab,ti OR 'invasive pneumococcal disease':ab,ti OR 'meningitis':ab,ti	236,043
4	Results of search no. 2 OR 3	4,243,047
5	'united states':ab,ti OR 'north america':ab,ti	306,077
6	Results of search no. 4 AND 5	110,059
7	Results of search no. 1 AND 6	611
8	'clinical trial'/de OR 'controlled clinical trial'/de OR 'double blind procedure'/de OR 'model'/de OR 'nonhuman'/de OR 'phase 3 clinical trial'/de OR 'practice guideline'/de OR 'randomized controlled trial'/de OR 'randomized controlled trial (topic)'/de	7,890,121
9	Results of search no. 7 NOT 8	396
10	Results of search no. 9 AND [english]/lim AND [1999–2019]/py	340

*As of 2019 May 21.

Appendix Table 4. Criteria for study selection in literature review of pneumococcal conjugate vaccines

Characteristic	Criteria
Population	Humans of any age
Interventions	PCV7 or PCV13
Comparators	No vaccination (or historical data)
Outcomes	Incidence and death rates of diseases preventable by PCVs†
Study design	Observational

*PCV, pneumococcal conjugate vaccine; PCV7, 7-valent pneumococcal conjugate vaccine; PCV13, 13-valent pneumococcal conjugate vaccine.
†I.e., otitis media, pneumonia, pneumococcal disease, invasive pneumococcal disease, and meningitis.

Appendix Table 5. Additional limits for inclusion in the literature review of pneumococcal conjugate vaccines

Characteristic	Criteria
Population	Pediatric population only (<19 y of age)
Interventions	No additional criteria
Comparators	No additional criteria
Outcomes	Incidence (including serotype distribution)<br>Prevalence/frequency (including serotype distribution)<br>Deaths<br>Costs<br>Hospitalizations, outpatient healthcare visits, and emergency room visits

Appendix Table 6. Population of young children, United States, 1997–2019*

	Age, y		
Year	<1	1	2–4
1997	3,751,141	3,755,827	11,725,703
1998	3,762,809	3,768,112	11,614,002
1999	3,795,762	3,784,001	11,555,781
2000	3,855,956	3,798,691	11,523,646
2001	4,012,658	3,855,407	11,430,152
2002	3,951,461	4,004,674	11,473,057
2003	3,975,871	3,936,139	11,680,436
2004	4,014,258	3,953,063	11,818,564
2005	4,004,393	3,987,032	11,925,975
2006	4,041,738	3,972,124	11,925,021
2007	4,147,997	4,002,215	11,975,750
2008	4,132,735	4,100,756	12,037,636
2009	4,003,587	4,078,797	12,162,134
2010	3,952,444	3,951,024	12,297,061
2011	3,963,092	3,966,225	12,193,786
2012	3,926,570	3,977,550	12,071,946
2013	3,931,258	3,942,696	11,975,260
2014	3,954,786	3,948,892	11,968,675
2015	3,983,965	3,973,192	11,960,948
2016	3,955,192	4,003,594	11,963,579
2017	3,893,945	3,973,803	12,024,219
2018	3,848,208	3,912,900	12,049,167
2019†	3,848,208	3,912,900	12,049,167

*(*22*).
†2019 values imputed based on 2018 data.

Appendix Table 7. Incidence of invasive pneumococcal disease in study on pneumococcal conjugate vaccines, United States, 1997–2019*

	<1 y		1 y		2–4 y	
Year‡	Cases	Incidence†	Cases	Incidence†	Cases	Incidence†
1997	320	142.9	396	178.7	208	31
1998	397	165.3	483	202.5	265	36.9
1999	420	162.7	523	205.4	254	33.2
2000	394	147.8	433	161.6	242	30.1
2001	187	52.3	232	68.6	275	27.5
2002	148	38.5	121	31.5	172	15.8
2003	155	41.3	134	35.7	144	13.1
2004	153	37	130	31.7	148	12.3
2005	153	36.2	151	35.6	155	12.3
2006	142	33.8	156	36.7	149	11.7
2007	180	40.51	142	32.39	168	13.03
2008	164	36.7	139	31.3	154	11.8
2009	165	37.1	144	32.1	182	13.6
2010	142	34.2	112	26.6	171	13.1
2011	88	21	71	16.9	108	8.3
2012	65	15.7	57	13.6	75	5.9
2013	61	14.7	63	15.1	86	6.8
2014	66	15.9	43	10.3	80	6.3
2015	77	18.4	54	12.9	65	5.1
2016	56	13.3	67	5.3	78	1.4
2017	48	11.6	33	7.9	66	5.2
2018§	48	11.6	33	7.9	66	5.2
2019§	48	11.6	33	7.9	66	5.2

*(*12*).
†Incidence per 100,000 children.
‡7-valent pneumococcal conjugate vaccine introduced 2000; 13-valent pneumococcal vaccine introduced 2010.
§2018 and 2019 values imputed based on 2017 data,

Appendix Table 8. Average incidence of invasive pneumococcal disease among young children, United States, 1997–2009*

Era	Pre-PCV7 (1997–1999)			PCV7 (2002–2009)		
Age, y	<1	1	2–4	<1	1	2–4
Incidence†	157	196	34	38	33	13

*PCV7 introduced 2000. PCV7, 7-valent pneumococcal conjugate vaccine.
†Incidence per 100,000 children.

Appendix Table 9. Invasive pneumococcal disease incidence among children <5 years of age, United States, 1997–2019*

Year	All†	PCV13 serotypes†	Non-PCV13 serotypes†
1997‡	81.67	75.08	5.74
1998	94.73	88.00	6.73
1999	92.94	88.00	4.94
2000	79.81	72.00	7.81
2001	40.87	34.00	6.87
2002	23.65	16.00	7.65
2003	23.36	14.00	9.36
2004	21.19	12.00	9.19
2005	21.77	13.00	8.77
2006	21.16	13.00	8.16
2007	22.54	15.00	7.54
2008	20.82	13.00	7.82
2009	21.97	15.00	6.97
2010	19.87	13.00	6.87
2011	12.50	4.00	8.50
2012	9.36	2.00	7.36
2013	10.01	2.00	8.01
2014	9.01	2.00	7.01
2015	9.32	2.00	7.32
2016	4.55	2.00	2.55
2017§	6.99	2.00	4.99
2018§	6.98	2.00	4.98
2019§	6.98	2.00	4.98

*(*12*).
†Incidence per 100,000 children.
‡1997 values imputed by extrapolating incidence using the ratio of total incidence change during 1997–1998, weighted by 1997 age distribution.
§2017–2019 values imputed based on Active Bacterial Core surveillance (*12*) serotype distribution data for 2016 applied to 2017–2019 incidence.

Appendix Table 10. Average incidence and distribution of invasive pneumococcal disease before introduction of PCV7, United States, 1997–1999*

Measurement	All	PCV13 serotypes	Non-PCV13 serotypes
Incidence†	89.78	83.69	5.80
Distribution, %	100	93.17	6.83

*PCV7 introduced 2000; PCV13 introduced 2010. PCV7, 7-valent pneumococcal conjugate vaccine; PCV13, 13-valent pneumococcal conjugate vaccine.
†Incidence per 100,000 children.

Appendix Table 11. Percent distribution of invasive pneumococcal disease syndromes, United States, 1997–2019*

	<2 y					2–4 y				
Year	Meningitis	Bacteremia	Bacteremic pneumonia/ empyema	Sepsis	Other	Meningitis	Bacteremia	Bacteremic pneumonia/ empyema	Sepsis	Other
1997†	6.68	58.57	20.05	0.00	14.70	5.37	59.09	28.10	0.00	7.44
1998†	6.68	58.57	20.05	0.00	14.70	5.37	59.09	28.10	0.00	7.44
1999†	6.68	58.57	20.05	0.00	14.70	5.37	59.09	28.10	0.00	7.44
2000‡	6.68	58.57	20.05	0.00	14.70	5.37	59.09	28.10	0.00	7.44
2001	8.13	54.31	22.73	0.00	14.83	4.73	53.82	35.27	0.00	7.27
2002	10.37	48.52	24.44	0.00	17.04	2.98	55.36	34.52	0.00	7.14
2003	11.00	51.89	23.37	0.00	15.12	0.69	48.28	42.76	0.00	8.28
2004	8.48	55.12	25.09	0.00	11.66	5.41	41.89	43.92	0.00	8.78
2005	11.84	54.28	24.34	0.00	11.18	6.41	40.38	47.44	0.00	6.41
2006	9.40	51.01	28.19	0.00	12.08	4.64	41.72	50.99	0.00	4.64
2007	10.67	49.09	26.83	0.00	14.02	2.98	36.90	50.60	0.00	10.12
2008	10.78	48.04	26.14	0.00	15.69	5.84	32.47	55.19	0.00	7.79
2009	7.14	46.43	29.55	0.00	17.53	2.66	38.30	49.47	0.00	9.57
2010‡	10.63	44.88	31.10	0.00	14.57	5.23	30.81	51.16	0.00	13.95
2011	15.19	43.04	23.42	0.00	20.25	5.61	38.32	44.86	0.00	12.15
2012	11.57	53.72	18.18	0.00	16.53	1.33	53.33	30.67	0.00	14.67
2013	7.69	57.26	23.93	0.00	12.82	10.47	40.70	39.53	0.00	10.47
2014	18.35	42.20	23.85	0.00	15.60	7.50	43.75	41.25	0.00	7.50
2015	16.03	47.33	19.08	0.00	19.08	3.03	43.94	42.42	0.00	10.61
2016	18.92	45.05	18.02	0.00	18.02	7.46	35.82	40.30	0.00	16.42
2017	12.20	43.90	25.61	0.00	20.73	12.12	45.45	36.36	0.00	7.58
2018§	12.20	43.90	25.61	0.00	20.73	12.12	45.45	36.36	0.00	7.58
2019§	12.20	43.90	25.61	0.00	20.73	12.12	45.45	36.36	0.00	7.58

*(*12*).
†1997–1999 values imputed based on 2000 values.
‡7-valent pneumococcal conjugate vaccine introduced 2000; 13-valent pneumococcal vaccine introduced 2010.
§2018 and 2019 values imputed based on 2017 values.

Appendix Table 12. Incidence of healthcare visits for otitis media, United States, 1997–2019*

Year	<2 y	2–<5 y	<5 y
1997	122	48	78
1998	122	48	78
1999†	113	46.25	73.25
2000†‡	103	44.5	68.5
2001†	93.5	42.75	63.75
2002	84	41	59
2003	84	41	59
2004	84	41	59
2005	84	41	59
2006	84	41	59
2007	84	41	59
2008	84	41	59
2009	84	41	59
2010‡§	74	37.5	52.5
2011	64	34	46
2012	64	34	46
2013	64	34	46
2014¶	64	34	46
2015¶	64	34	46
2016¶	64	34	46
2017¶	64	34	46
2018¶	64	34	46
2019¶	64	34	46

*Incidence per 100 children reported as the average incidence for the pre PCV period (1998-1999), PCV7 period (2002-2009), and PCV13 period (2011-2013) (*23*).
†1999–2001 values imputed based on a linear interpolation of 1998–2002 values.
‡7-valent pneumococcal conjugate vaccine introduced 2000; 13-valent pneumococcal vaccine introduced 2010.
§2010 values imputed based on a linear interpolation of 2009–2011 values.
¶2014–2019 values imputed based on 2013 values, assuming no further change in incidence.

Appendix Table 13. Average incidence of healthcare visits for otitis media, United States, 1997–2019*

Era	Pre-PCV (1997–1999)			PCV7 (2002–2009)		
Age, y	<2	2–5	<5	<2	2–5	<5
Cases	122	48	78	84	41	59

*(*23*). PCV7 introduced 2000. PCV7, 7-valent pneumococcal conjugate vaccine.

Appendix Table 14. Effect of PCVs on estimated hospitalizations for pneumonia, United States, 1997–2019*

	Observed				Expected without vaccination			
Era	Grijalva et al. (*26*)	Foote et al. (*25*)	Simonsen et al. (*24*)	Tong et al. (*28*)†	Grijalva et al. (*26*)	Foote et al. (*25*)	Simonsen et al. (*24*)	Tong et al. (*28*)†
Pre-PCV (1997–1999)	425,792	525,675	339,474	NA	425,792	525,675	339,474	NA
PCV7 (2000–2009)	1,106,634	1,336,673	959,543	597,479	1,481,474	1,822,591	1,182,154	597,479
PCV13 (2010–2019)	NA	NA	NA	382,182	NA	NA	NA	598,484

*PCV7 introduced 2000; PCV13 introduced 2010. NA, not applicable; PCV, pneumococcal conjugate vaccine; PCV7, 7-valent pneumococcal conjugate vaccine; PCV13, 13-valent pneumococcal conjugate vaccine.
†Analysis includes only index cases.

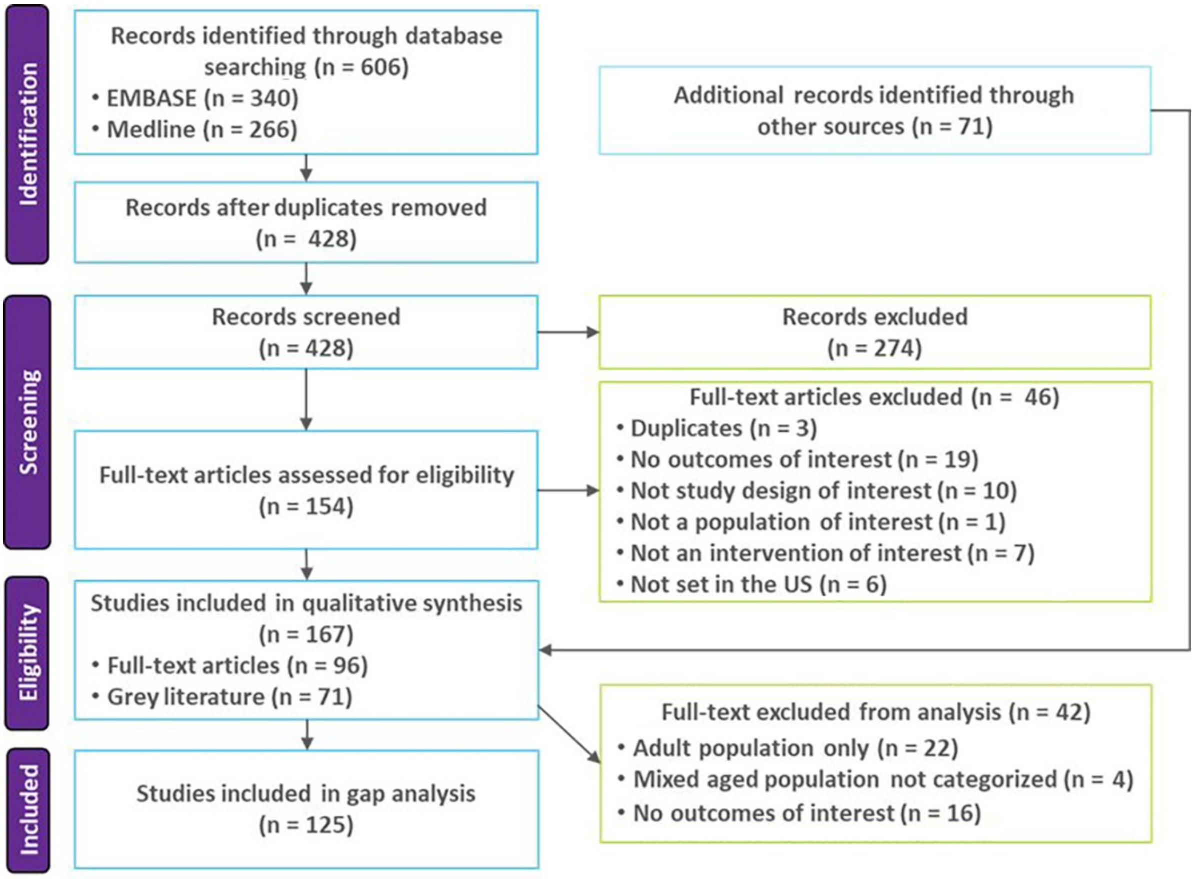

Appendix Figure 1. Study design of systematic literature review on pneumococcal conjugate vaccines, United States, 1997–2019.

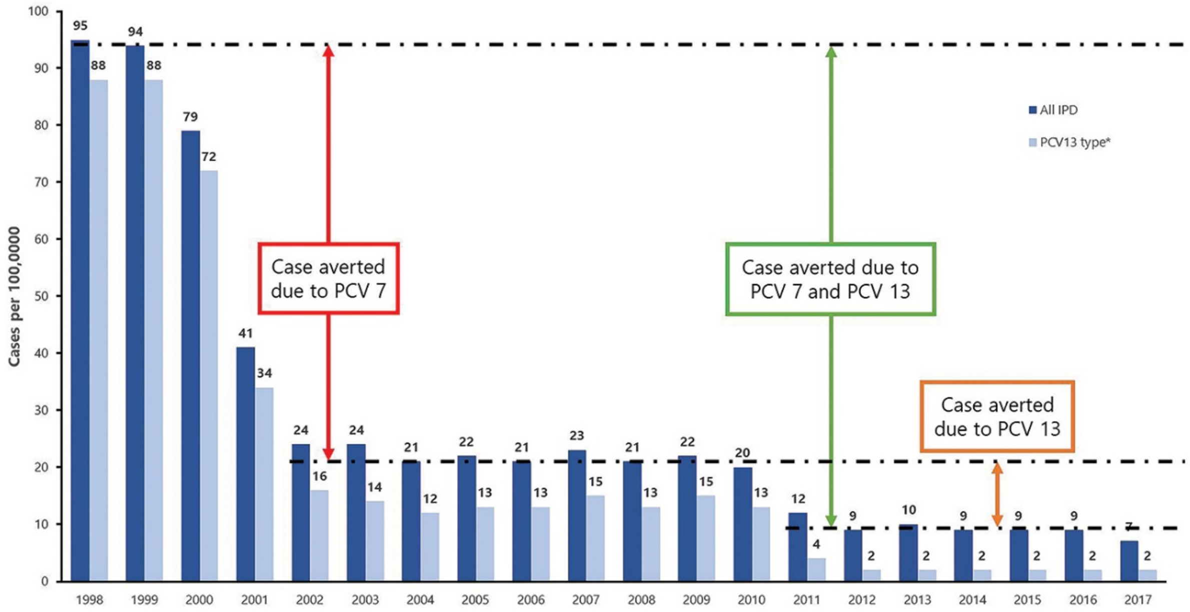

Appendix Figure 2. Incidence of invasive pneumococcal disease among children <5 years of age, United States, 1998–2017. Figure adapted from US Centers for Disease Control and Prevention (https://www.cdc.gov/abcs/reports-findings/survreports/spneu-types.html). Dark blue bars indicate all cases of invasive pneumococcal disease; light blue bars indicate cases caused by serotypes contained in PCV13. Red arrows indicated cases averted by PCV7; green arrows indicate cases averted by PCV7 and PCV13; orange arrows indicate cases averted by PCV13. PCV13 prevents disease caused by *Streptococcus pneumoniae* serotypes 1, 3, 4, 5, 6A, 6B, 7F, 9V, 14, 18C, 19A, and 23F. PCV7, 7-valent pneumococcal conjugate vaccine; PCV13, 13-valent pneumococcal conjugate vaccine.

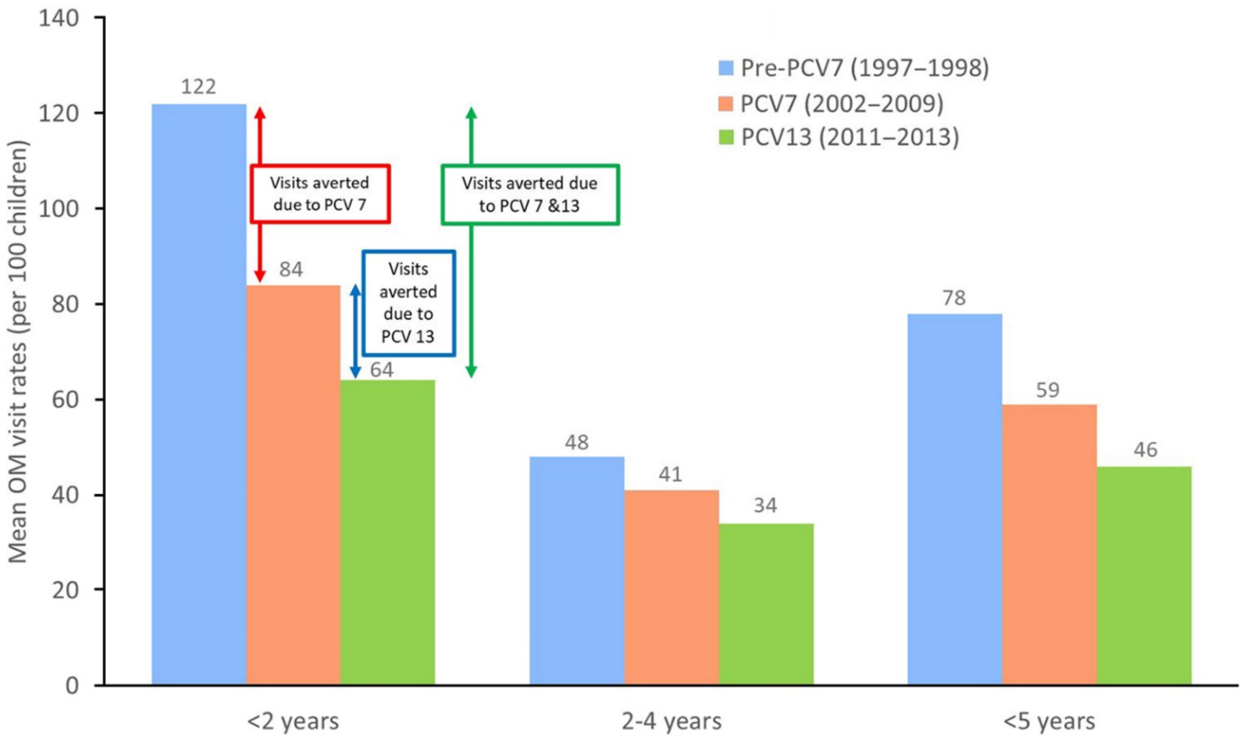

Appendix Figure 3. Mean healthcare visits for otitis media averted by PCVs, United States, 1997–2013. Incidence is per 100 children. Blue bars indicate pre-PCV era (1997–1999), orange bars indicate PCV7 era (2002–2009); green bars indicate PCV13 era (2011–2013). Red arrows indicated cases averted by PCV7; green arrows indicate cases averted by PCV7 and PCV13; blue arrows indicate cases averted by PCV13. PCV7, 7-valent pneumococcal conjugate vaccine; PCV13, 13-valent pneumococcal conjugate vaccine.

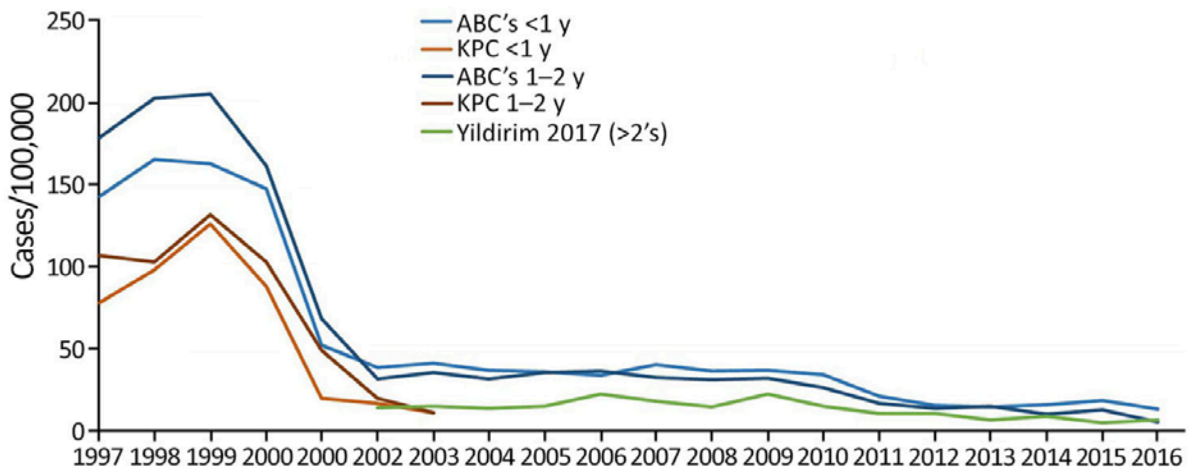

Appendix Figure 4. Validation of findings on invasive pneumococcal disease incidence, United States, 1997–2016. Findings were based on ABCs data (*12*) and validated against Black et al. (*30*) and Yildrim et al. (*31*). Colored lines indicate data sources and populations. ABCs, Active Bacterial Core Surveillance (*12*); IPD, invasive pneumococcal disease; KP, Kaiser Permanente (*30*).
